# Consultative meeting that examined alignment and discrepancies between health facility and household survey data in Malawi

**DOI:** 10.1186/s12936-019-3050-1

**Published:** 2019-12-09

**Authors:** Katherine E. Battle, Austin Gumbo, Gracious Hamuza, Collins Kwizombe, Akuzike Tauzi Banda, Steven Chipeta, Mphatso D. Phiri, Blessings Kamanga, Jacob Kawonga, Taonga Mafuleka, Ashley Malpass, Phinias Mfune, Mathews Mhango, Lumbani Munthali, Godfrey Silungwe, Memory Siwombo, Haroon Twalibu, Allison Zakaliya, Michael Kayange, Cameron Taylor

**Affiliations:** 1 Malaria Atlas Project, Big Data Institute, University of Oxford, Old Road Campus, Oxford, OX3 7LF UK; 2 National Malaria Control Programme, Ministry of Health, Lilongwe, Malawi; 3 U.S. President’s Malaria Initiative, United States Agency for International Development, Lilongwe, Malawi; 4 District Health Officer, Dowa, Malawi; 5 Malawi-Liverpool-Wellcome Trust Clinical Research Programme, Blantyre, Malawi; 6 Kuunika Project, Lilongwe, Malawi; 7 Central Monitoring Evaluation Division, Lilongwe, Malawi; 8 District Health Officer, Karonga, Malawi; 9 District Health Officer, Ntchisi, Malawi; 10 District Health Officer, Mzimba South, Malawi; 11 District Health Officer, Phalombe, Malawi; 12 Organized Network of Services for Everyone’s (ONSE) Health, Management Sciences for Health, Lilongwe, Malawi; 13 The DHS Program, ICF, Rockville, USA

**Keywords:** Malawi, The DHS program, Malaria Indicator Survey, DHIS2, Health Management Information System, Malaria Strategic Plan, Workshop

## Abstract

Malawi is midway through its current Malaria Strategic Plan 2017–2022, which aims to reduce malaria incidence and deaths by at least 50% by 2022. Malariometric data are available with health surveillance data housed in District Health Information Software 2 (DHIS2) and household survey data from two recent Malaria Indicator Surveys (MIS) and a Demographic and Health Survey (DHS). Strengths and weaknesses of the data were discussed during a consultative meeting in Lilongwe, Malawi in July 2019. The first 3 days included in-depth exploration and analysis of surveillance and survey data by 13 participants from the National Malaria Control Programme, district health offices, and partner organizations. Key indicators derived from both DHIS2 and MIS/DHS sources were analysed with three case studies, and presented to stakeholders on the fourth day of the meeting. Applications of the findings to programmatic decision-making and strategic plan evaluation were critiqued and discussed.

## Background

Malaria is a significant public health problem in Malawi that is the most frequently reported disease among children under age 5 at both outpatient departments and village clinics. Each year, approximately six million malaria cases account for 30% of all outpatient visits at health facilities, 34% of inpatient hospital admissions, and 2967 malaria-related hospital deaths, according to Malawi’s 2018 Health Management Information System (HMIS). In Malawi, the entire population is at risk of malaria infection and the incidence of malaria was 393 per 1000 in 2018, according to HMIS data.

The transmission of malaria in Malawi is perennial with the peak number of cases occurring between January and May, which is after the beginning of the annual rains. High burden areas are located in the warmer, more humid low-lying areas (lakeshore, Shire river valley, and central plain), while there is lower risk in the highland areas of Rumphi, Mzimba, Chitipa, and the Kirk Range [1]. *Plasmodium falciparum* is the most common species of malaria, which accounts for 98% of infections and all cases severe disease and death [2]. In the 2017 Malaria Indicator Survey (MIS), the prevalence of malaria parasites (by microscopy) in children under age 5 ranged from 11% in the Northern region to 26% in the Central and Southern regions, with the national average of 24% [3].

In the national Malaria Strategic Plan (MSP) for 2017–2022, Malawi, through the National Malaria Control Programme (NMCP), aims to reduce the incidence of malaria from 386 per 1000 population in 2015 to 193 per 1000 by 2022; and to reduce malaria deaths from 23 per 100,000 population in 2015 to 12 per 100,000 by 2022 [4]. The NMCP plans to achieve these goals by implementing efficacious interventions, such as long-lasting insecticide-treated nets (LLINs), prompt and effective case management, and intermittent preventive therapy in pregnancy (IPTp).

Specific objectives related to LLINs, case management, and IPTp in the 2017–2022 MSP are achieving universal LLIN access, which is one net for every two individuals in a household; ensuring that 95% of suspected malaria cases are tested and 100% of confirmed cases are treated with the recommended anti-malarial drugs, such as artemisinin-based combination therapy (ACT); and increasing the uptake of three or more doses of IPTp in pregnant women from 12% in 2014 to 60% by 2022. Other MSP objectives include promoting social behaviour change and communication that enhances community uptake of interventions; minimizing ACT stock-outs; strengthening surveillance, monitoring and evaluation (M&E), operational research; and improving data quality and accuracy.

The District Health Information Software 2 (DHIS2) (https://www.dhis2.org/) platform was implemented in Malawi to strengthen data management at the district and national levels by improving data capture and storage for programme and service delivery units in the public health sector. The platform was introduced with technical support from the University of Oslo, in collaboration with the University of Malawi College of Medicine, in 2011 and was adopted by the Ministry of Health and Population through the enactment of the Health Information Systems Policy in 2015.

In addition to robust routine surveillance data through DHIS2, Malawi has also conducted household surveys with the technical assistance of The Demographic and Health Survey (DHS) Program in Rockville, MD, USA, which is funded by the United States Agency for International Development (USAID). The most recent surveys include two Malaria Indicator Surveys (MIS), conducted in 2014 and 2017, and a DHS in 2015–2016. These nationally representative surveys gather data on indicators of the prevalence, treatment, and control of malaria.

A recent analysis examined the use of malaria M&E data to track progress and refine the targets in the MSP [5]. This study found that although targets had not been achieved, future MSP targets were set at the same or a higher value. The authors attribute this to the insufficient use of data. The availability of a digital routine surveillance platform and multiple household surveys in Malawi presents an opportunity for the NMCP to use complementary data sources to evaluate progress towards the MSP targets [4, 6]. Since there is little evidence in the literature that these data sources are being used together for strategic decision making [7], a Malaria Data Consultative Meeting was planned to determine how to leverage these data sources for programmatic support.

## Meeting structure and objectives

The aims of the meeting focused on the analysis of malaria indicators from the facility-based DHIS2 data and the household-based data from the 2014 Malawi MIS, the 2015–2016 Malawi DHS, and the 2017 Malawi MIS. The meeting was held in mid-July before the MSP review at the end of July. Meeting participants reviewed the 2017–2022 Malawi MSP to identify opportunities for complementary information from household survey data and to refine and inform DHIS2 data being used to evaluate MSP targets. This review led to the creation of case studies that would be used to examine situations when household and DHIS2 data sources provided discrepant results and when these sources could be used together to provide a more complete picture of existing malaria transmission and control.

The stated aims of the meeting were that, by the end of the meeting, participants would be able to:

- Describe the purpose/rationale of health facility indicators and household survey indicators;
- Discuss the methodological considerations and issues for interpreting health facility and household survey indicators;
- Describe how household survey indicators can complement health facility data to fill gaps;
- Identify areas of discrepancy between the health facility and household survey indicators;
- Investigate the reasons for the discrepancies between health facility and household survey indicators;
- Document the discrepancies and interpretation of these discrepancies among indicators measured by health facility and survey data in a case study.

The first part of the Malaria Data Consultative Meeting included a 3-day data exploration meeting designed for malaria data experts to explore the available indicators and produce graphics that would highlight similarities and differences between the DHS/MIS and health facility data. During this meeting, participants: (i) reviewed malaria M&E data sources; (ii) discussed the strengths and weaknesses of these data sources; (iii) reviewed the main household and facility-based malaria indicators; (iv) identified indicators from DHS/MIS surveys that complement the health facility data; (v) checked health facility-based data for external consistency with the DHS/MIS data; (vi) compared indicators to targets identified in the 2017–2022 MSP; and (vi) drafted three case studies that compared DHS/MIS and health facility data. In total, 13 individuals participated in the data exploration meeting (6—NMCP, 2-Ministry of Health (MoH), 2—Kuunika Project, 1—Central Monitoring Evaluation Division (CMED), 1—The DHS Program, and 1—Malaria Atlas Project).

The Malaria Data Consultative Meeting then included a 2-day presentation of results. This meeting was designed for a larger group of stakeholders who would discuss the findings of the data exploration meeting and comment on the case studies. The first day of the results presentation included oral presentations of the three case studies and a roundtable discussion, while the second day included a structured writing workshop focused on assembling the first draft of the meeting report. A total of 23 individuals participated in the results meeting.

## Identification of case study indicators

A list of 45 core indicators was assembled from the Malawi MSP and the World Health Organization (WHO) Guidance for Malaria Programme Managers [6, 8]. The selected indicators were metrics gathered from routine DHIS2 or household surveys that are commonly used to evaluate the success of malaria control programmes focused on vector control, chemoprevention, case detection, diagnostic testing, and treatment. Key meeting activities included reviewing the list of metrics and identifying which indicators could be measured with both sources. There was a small amount of overlap, which is to be expected given that the data sources are not designed to replicate indicators, but rather to complement each other. For the six indicators found in both data sources, participants explored how the metrics could be disaggregated by geography (region or district) or population (pregnant women or children under age 5). Three indicators that were strong candidates for the case studies, based upon the number of data collection years in the DHIS2 and household surveys were distribution of LLINs to pregnant women through antenatal clinics (ANCs), diagnostic testing rates in children under age 5, and uptake of two or more doses of IPTp by pregnant women. The indicators were restricted to children under age 5 and pregnant women for comparability between data sources, but these key risk groups were also highlighted in the MSP targets.

### Case study 1: LLIN distribution through antenatal clinics

Based on the success of net use, the distribution and promotion of LLINs is the primary malaria prevention intervention described in the Malawi MSP 2017–2022. Strategies for LLIN distribution in Malawi include free distribution to pregnant women through ANC visits and newborns at the time of delivery, as well as mass distribution campaigns every 3 years.

Since 2006, the NMCP has provided free routine distribution of LLINs to pregnant women during their first ANC visit. This information is included in the DHIS2 system in order to monitor the number of nets distributed over time. Within the household surveys, the household questionnaire includes detailed information about each LLIN in the household, including where each net was obtained. This has allowed for a comparison of data between LLINs distributed through ANC in the routine data and households owning a net from ANC in the survey data.

Table 1 shows the indicators examined in this case study. The population denominator for the health facility data included pregnant women who attended ANC. To obtain the most comparable metric, the household survey study population was restricted to women who had a live birth in the 2 years before the survey. The presentation of this case study emphasized that although the denominators differed, the two indicators can be examined together to assess trends in LLIN distribution at ANC.

**Table 1:** Available data for the examination of LLIN distribution through ANC

Data source	Available data	Indicator	Numerator	Denominator
DHIS2	2014–2018	Proportion of pregnant women who received an LLIN at ANC	Number of pregnant women who received an LLIN at ANC	Total number of pregnant women who attended ANC
Household survey data	2015–16 Malawi DHS and 2017 Malawi MIS	Proportion of women who had a live birth in the last 2 years and live in in a household that received an LLIN from ANC	Number of women in households that received an LLIN from ANC	Total number of women who had a live birth in the past 2 years

**Fig. 1:**
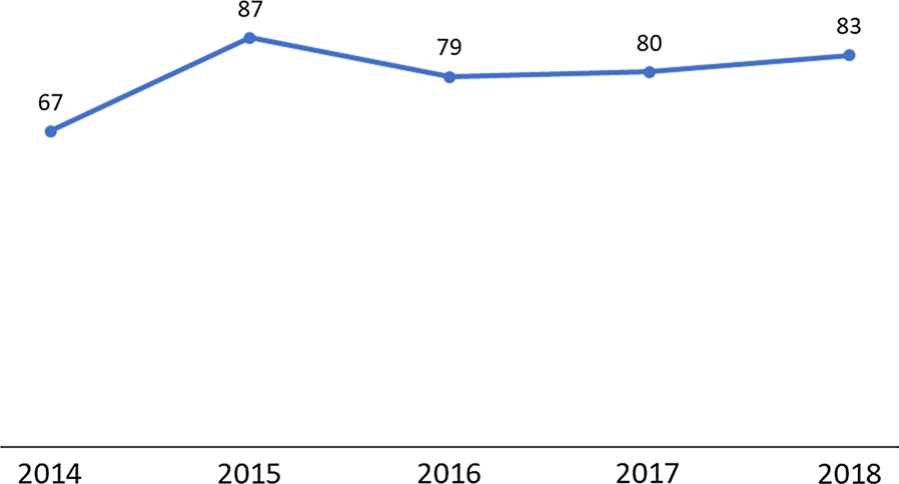
Percentage of women who received LLINs at ANC from 2014–2018 (Source: DHIS2 2014–2018)

In the DHIS2 data from 2014–2018, the percentage of women who received LLINs at ANC increased from 67% in 2014 to 87% in 2015. Distribution of LLINs to pregnant women at ANC then decreased to 79% in 2016, before it increased four percentage points to 83% in 2018 (Fig. 1). The fluctuation in the distribution of LLINs at ANC was attributed to data management issues such as poor documentation (providing an LLIN to pregnant women without recording) and stock-out of LLINs at ANCs.

**Fig. 2:**
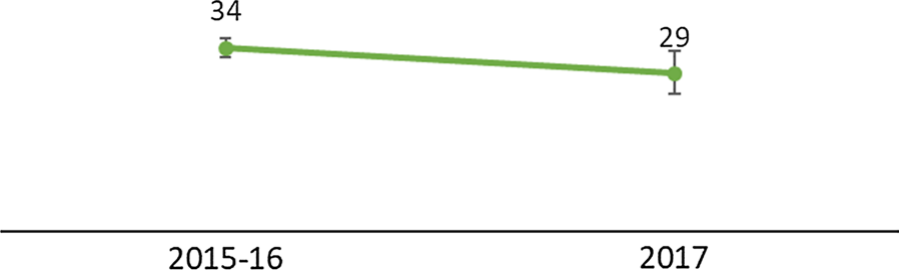
Among women who have had a live birth in the past 2 years, percentage of women in households that received an LLIN from ANC (Source: 2015–2016 Malawi DHS and 2017 Malawi MIS)

Information about LLINs obtained at ANC from household surveys was only available for the 2015–2016 Malawi DHS and the 2017 Malawi MIS. From 2015–2016 to 2017, the trend for women who had a child in the past 2 years living in households with an LLIN from ANC decreased from 34% (95% confidence interval [CI] 32.5%–35.9%) in 2015–16 to 29% (95% CI 25.5%–33.7%) in 2017 (Fig. 2).

Comparison of the DHIS2 and DHS/MIS data sources showed a decreasing trend during the years 2015–2017. Although the study populations (denominators) of the two datasets are different, the general trend is the same.

The difference in magnitude between the two data sources is explained by the fact that the DHIS2 data are recorded at the time of receiving the net at ANC. In contrast, household survey data are biased because the net ownership in households may have been obtained up to 2 years ago. The net received at ANC may no longer be functional, depending upon how long ago the woman gave birth to her child under age 2.

From 2014 to present, Malawi has made great strides towards achieving the national MSP targets that call for 85% of pregnant women to sleep under an LLIN by 2019 and 90% by 2021. Using these data, the NMCP will review data management of LLIN distribution at ANC, with the goal of improving data quality and determining areas where women are not receiving LLINs at ANC.

### Case study 2: testing rates

Effective malaria case management is a key component of the 2017–2022 MSP, which stipulates that at least 95% of suspected malaria cases will be tested and 100% of confirmed cases treated by 2022. The indicators in Table 2 were used to examine the progress made in using microscopy or rapid diagnostic tests (RDTs) to confirm a malaria diagnosis. From the DHIS2 data, testing rates were examined as the percentage of suspected malaria cases in children under age 5 that received a confirmatory test at a facility or village clinic. From the household survey data, rates of testing were examined using those children under age 5 who had a fever in the previous 2 weeks for whom advice or treatment was sought and who had blood taken from a finger or heel for malaria testing.

**Table 2:** Available data for the examination of malaria testing rates

Data source	Available data	Indicator	Numerator	Denominator
DHIS2	2014–2018	Percentage of suspected malaria cases in children under age 5 who received a confirmatory test at facility or village clinic	Number of suspected malaria cases in children under age 5 who received a confirmatory test	Total number of suspected cases in children under age 5 at facility or village clinic
Household survey data	2014 Malawi MIS and 2017 Malawi MIS	Percentage of children under age 5 with fever in the previous 2 weeks for whom advice or treatment was sought and who had blood taken from a finger or heel for testing	Number of children under age 5 with fever in the previous 2 weeks for whom advice or treatment was sought and who had blood taken from a finger or heel for testing	Total number of children under age 5 with fever in the previous 2 weeks for whom advice or treatment was sought

**Fig. 3:**
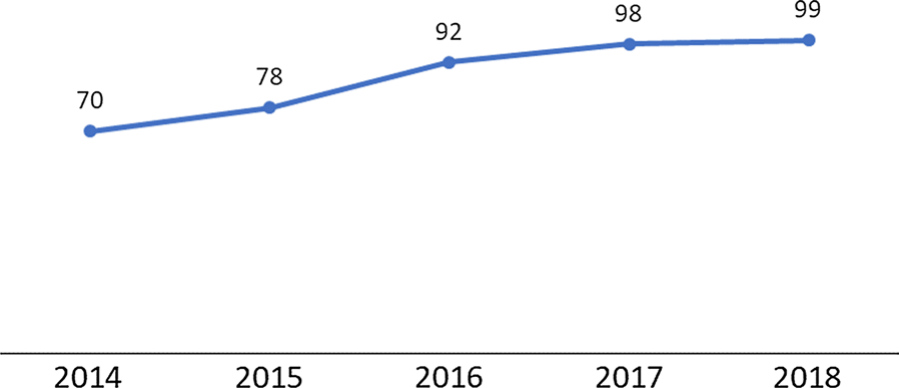
Percentage of suspected malaria cases in children under age 5 that received a confirmatory test at facility and village clinics from 2014–2018 Source: DHIS2 2014–2018

**Fig. 4:**
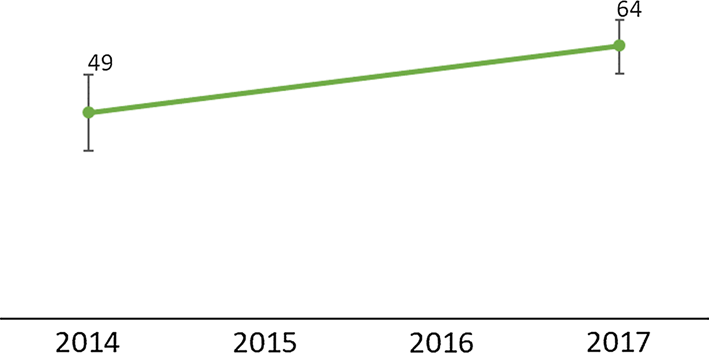
Percentage of children under age 5 with fever in the previous 2 weeks for whom advice or treatment was sought and who had blood taken from a finger or heel for testing (Source: DHIS2 2014–2018)

There was an overall increase in testing rates across the country between 2014 and 2018, as shown by testing rates from the DHIS2 and household survey results below. The DHIS2 data had a higher percentage point increase (29% in Fig. 3), compared to the household survey with a 15 % point increase between 2014 and 2017 (Fig. 4). The DHIS2 data had higher coverage rates compared to the household survey data. The estimated testing rates from routine data lie outside the 95% confidence interval estimated from the household data survey results for the years 2014 and 2017, which suggests a significant difference in the values. These differences were attributed primarily to recall bias among the survey respondents as health cards/passports are not used for heel or finger sticks.

**Fig. 5:**
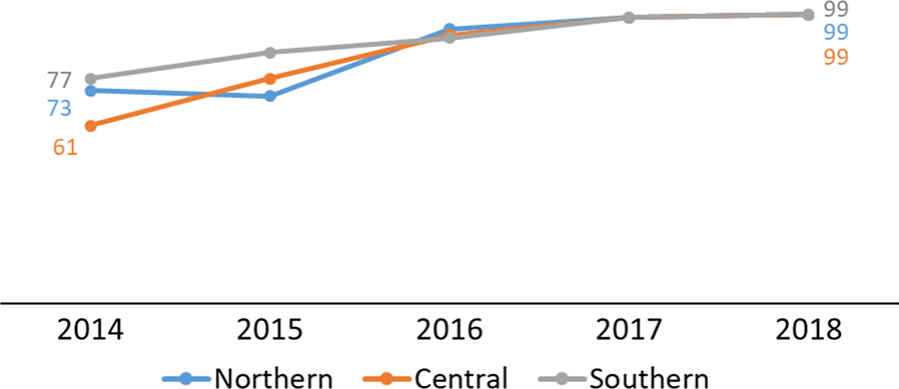
Percentage of suspected malaria cases in children under age 5 who received a confirmatory test at facility and village clinics from 2014–2018 by region (Source: 2014 and 2017 Malawi MIS)

**Fig. 6:**
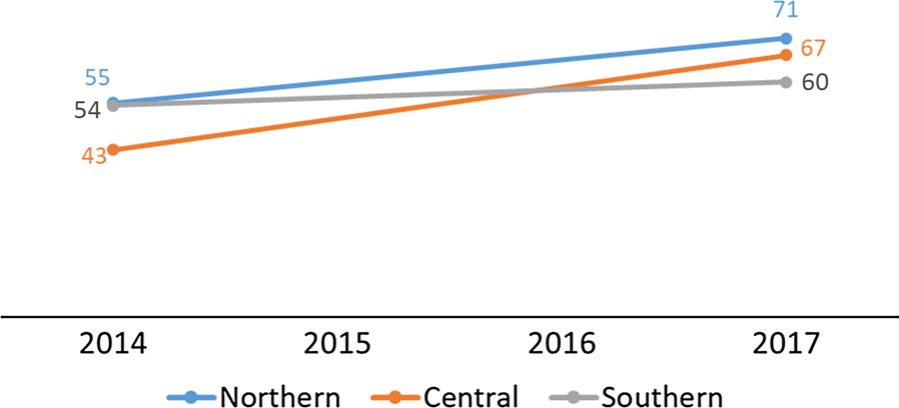
Percentage of children under age 5 with fever in the previous 2 weeks for whom advice or treatment was sought and who had blood taken from a finger or heel for testing by region (Source: DHIS2 2014–2018)

Testing rates across the Northern, Central, and Southern regions of Malawi showed consistent improvement between 2016 and 2018 and converged at 99% in 2017 and 2018 (DHIS2) (Fig. 5). The household survey data showed similar results. By region, changes in testing rates in the Northern and Southern regions were not significantly different with increases from 55% (95% CI 34%–74%) in 2014 to 71% (95% CI 58%–81%) in 2017 in the Northern region, and from 54% (95% CI 38%–69%) in 2014 to 60% (95% CI 48%–71%) in 2017 in the Southern region. The change was significant in the Central region from 43% (95% CI 31%–55%) in 2014 to 67% (95% CI 59%–74%) in 2017 (Fig. 6). The programme performed above the MSP strategic target of 90% in 2016, although it had slightly missed the 2014 and 2015 targets as shown in Table 3.

**Table 3:** Testing rates, malaria strategic plan targets, and reported rates from DHIS2 data sources [4, 6]

Indicator	2014 (%)	2015 (%)	2016 (%)	2017 (%)	2018 (%)	2022 (%)
Percentage of suspected malaria cases that received parasitological test						
MSP target	74	82	90	85	87	95
Actual achievement (DHIS2)	70	78	92	98	99	

The use of RDTs in village clinics was not standard in Malawi until 2016, and this may have contributed to the lower testing rates in 2014 and 2015. The increase between 2016 and 2018 was attributed to the 2016 introduction of malaria RDTs at the community level. The programme performed above MSP strategic targets in 2017 and 2018. The joint results of the DHIS2 and household survey data serve as a reminder of the importance of sustaining gains achieved by maintaining continuous availability of malaria RDTs, which will be considered at the time of the midterm MSP review.

### Case study 3: uptake of IPTp2**+**

The 2017–2022 MSP identifies access to IPTp with sulfadoxine-pyrimethamine (SP/Fansidar) for pregnant women as a key control target. The MSP specifies three or more doses (IPTp3+), although IPTp+was not added as an indicator to the DHIS2 until 2019. Therefore, to examine uptake of IPTp, pregnant women who received two or more doses (IPTp2+) were examined. As specified in Table 4 below, the indicator, based on DHIS2 data, shows the proportion of the total number of pregnant women registered at ANC who received two or more doses of SP at their ANC visits. From the 2014 and 2017 MIS and the 2015–2016 DHS data, the proportion of women who received IPTp2+was calculated from the number of women who delivered a live child in the previous 2 years.

**Table 4:** Available data for the examination of access to two or more doses of IPTp

Data source	Available data	Indicator	Numerator	Denominator
DHIS2	2014–2018	Proportion of women who received two or more doses of SP during ANC visits	Number of women who received two or more SP doses at ANC	Total number of pregnant women registered at ANC
Household survey data	2015–2016 Malawi DHS and 2014 2017 Malawi MIS	Proportion of women who received two or more doses of SP and received at least one dose during an ANC visit during their last pregnancy that led to a live birth in the previous 2 years	Number of women who received two or more doses of SP and received at least one dose during an ANC visit during their last pregnancy that led to a live birth in the previous 2 years	Total number of women surveyed who delivered a live child in the previous 2 years

**Fig. 7:**
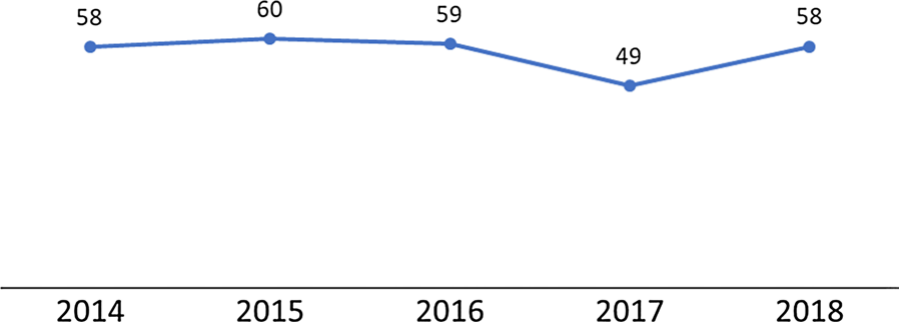
Percentage of pregnant women who received at least 2 doses of SP during ANC among the total number of new pregnant women registered at ANC (Source: 2014 and 2017 Malawi MIS)

**Fig. 8:**
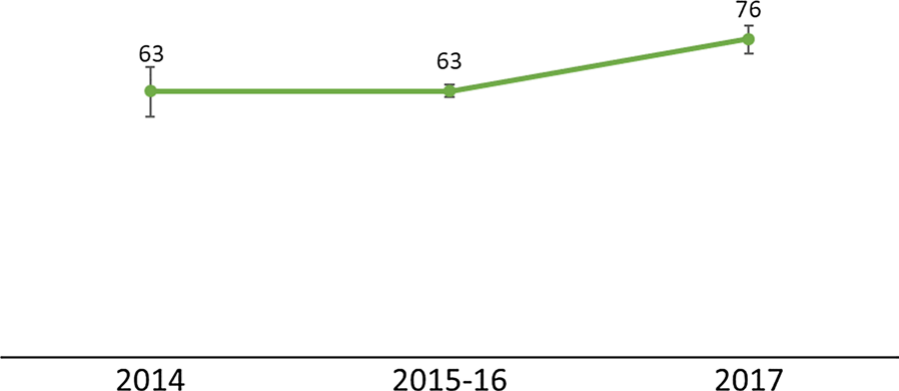
Percentage of women age 15–49 who received at least two doses of SP/Fansidar during their last pregnancy in the 2 years before the survey (Source: 2014 Malawi MIS, 2015–2016 Malawi DHS, and 2017 Malawi MIS)

As shown in Fig. 7, the pattern of uptake of IPTp2+was different for DHIS2 data compared to the household survey data (Fig. 8). Although routine surveillance data show variable progress in IPTp2+, the household survey data indicates steady progress. The potential causes for these differences include data quality issues with the routine surveillance values such as reporting completeness and accuracy. There may have also been some recall bias in the household survey data because of the retrospective questions about IPTp. For example, a woman may not recall the number of doses she received during her last pregnancy in the previous 2 years.

**Fig. 9:**
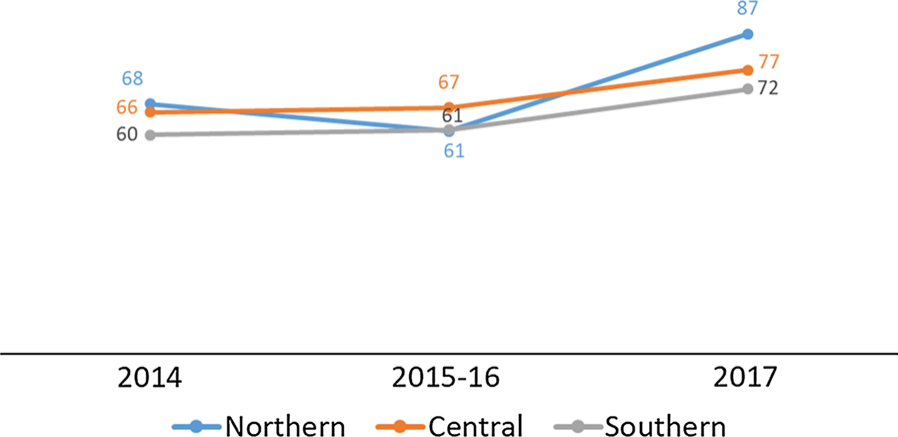
Percentage of women age 15–49 who received at least two doses of SP/Fansidar during their last pregnancy in the 2 years before the survey by region (Source: 2014 Malawi MIS, 2015–16 Malawi DHS, and 2017 Malawi MIS)

**Fig. 10:**
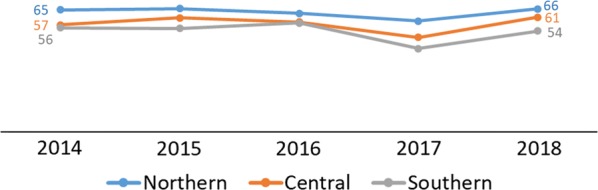
Percentage of pregnant women who received at least two doses of SP/Fansidar during ANC among the total number of new pregnant women registered at ANC by region (Source: DHIS2 2014–2018)

The data showed complementary patterns by region with the DHS/MIS and DHIS2 data showing higher uptake of IPTp2+ in the Northern region of Malawi as compared to the Southern and Central regions (Figs. 9 and 10). Regional differences were attributed to common determinants of health that vary among the regions such as literacy and socioeconomic factors [3]. The 2017 MSP target for IPTp2+ for 2017 was 70% [6]. According to the 2017 MIS, that target was surpassed, although the DHIS2 data show a 12% point deficit (Fig. 10).

## Discussion

After the presentation of each case study, there was an opportunity for discussion and feedback from the stakeholders present for the second portion of the data consultative meeting in Lilongwe. Participants identified additional data that would complement the results of the case studies and recommended next steps based on the MSP. This was programmatically relevant given the upcoming review of the MSP.

Some issues arose in the three case studies. The stakeholders reiterated that rather than comparing the absolute values of the indicators from the two data sources, the similarities (or discrepancies) in the trends should be the preferred focus. It is not possible to have the exact same denominator for each indicator (Tables 1, 2 and 4), which makes it difficult to compare values rather than trends. In addition, although routine data sources are subject to quality issues such as reporting completeness and the influence of commodity stockouts, survey data are also prone to recall or respondent bias. Finally, survey data may be able to capture the impact of the private sector more effectively than routine surveillance. Although some private sources of care are captured in DHIS2 (Christian Health Association of Malawi [CHAMs] facilities), many are not. Thus, it is not possible to quantify the percentage of care that is sought but is unavailable.

It was emphasized that although net use is included in the MSP, distribution is not. Completeness of distribution could be a benchmark so that access as well as use is assessed. Although net distribution at ANC and birth includes key risk groups, it would also be beneficial to organize other routine distribution points such as schools.

Both the DHIS2 and household survey data show improvement in the testing of suspected cases over time. According to the DHIS2 data, the MSP targets have been met. When all ages (instead of only children under age 5) were examined, testing rates exceeded 100%. This may reflect data quality issues that need to be addressed. It is also important to report testing rates and the status of commodity stocks, so that the root causes of low rates can be determined. Children under age 5 were the focus of the case study because it allowed for a more direct comparison with the household survey data. However, confirmation of all suspected cases should be a priority, as outlined in the MSP. There may be additional data sources to consider when assessing this metric, such as Logistics Management Information Systems (LMIS) or Multiple Indicator Cluster Surveys (MICS).

Assessment of IPTp showed some surprisingly low rates of uptake given that the indicator reflected two or more doses of SP during pregnancy and the recommendation is now three or more. However, many participants emphasized that IPTp doses are inherently dependent on the number of visits made to ANC and many women do not go to ANC until their second trimester of pregnancy, which limits the number of doses a woman can receive. Next steps include improving community engagement to break down barriers to IPTp uptake such as myths or superstitions associated with early ANC attendance and institutional barriers such as commodity availability (SP/Fansidar), as well as increasing access to care points by taking the services closer to communities, which the NMCP is currently piloting. A key point from the stakeholder discussions was that community awareness is a key factor in determining the levels of the indicators and that there needs to be greater focus on other determinants of health such as socioeconomic issues and education.

## Conclusions

Consistent evaluation of planned targets is needed as countries work to control malaria. Malawi has assembled a wealth of data that can serve as benchmarks to programmatic success. The adoption of the DHIS2 platform for routine surveillance of data has made facility and clinic-based data more readily accessible and reliable. Multiple household surveys conducted with standardized methodologies provide consistent indicators over time. Data from DHIS2 and household surveys have different strengths and weaknesses. Routine surveillance is subject to errors in reporting and completeness and is dependent on commodity stocks, but includes data from the entire country, which can be aggregated at subnational levels and followed longitudinally. Survey data are collected at a single point in time, are subject to recall bias, and do not provide subnational estimates, although the survey design allows surveys from different years and locations to be compared by a range of outcomes and predictors. The case studies presented in the meeting illustrate that routine data and household survey data can be used together to produce a more complete, unbiased picture of malaria in Malawi.

## Data Availability

All household survey data are available from The DHS Program: https://dhsprogram.com/What-We-Do/Survey-Search.cfm. Routine surveillance data housed in DHIS2 were shared by NMCP meeting participants for analysis during the workshop.

## Abbreviations

ACT: artemisinin-based combination therapy
ANC: antenatal clinic
CCM: Community Case Management
CHAM: Christian Health Association of Malawi
DHIS2: district health information system 2
DHO: district health office
DHS: demographic and health survey
HMIS: health management information system
IPTp: intermittent preventive therapy in pregnancy
LLIN: long-lasting insecticide-treated net
LMIS: logistics management information system
M&E: monitoring and evaluation
MICS: multiple indicator cluster survey
MIS: Malaria Indicator Survey
MoH: Ministry of Health
MSP: Malaria Strategic Plan
NMCP: National Malaria Control Programme
RDT: rapid diagnostic test
SP: sulfadoxine-pyrimethamine
USAID: United States Agency for International Development
WHO: World Health Organization

## References

[1] Kazembe LN, Kleinschmidt I, Holtz TH, Sharp BL (2006). Spatial analysis and mapping of malaria risk in Malawi using point-referenced prevalence of infection data. Int J Health Geogr.

[2] U.S. President’s Malaria Initiative (PMI). Malawi abbreviated malaria operational plan. 2019.

[3] National Malaria Control Programme, and ICF. Malawi Malaria Indicator Survey 2017. Lilongwe, Malawi, 2018.

[4] Programme National Malaria Control (2011). Malaria Strategic Plan 2011–2016.

[5] Andrada A, Herrera S, Yé Y (2019). Are new national malaria strategic plans informed by the previous ones? a comprehensive assessment of sub-Saharan African countries from 2001 to present. Malar J.

[6] Programme National Malaria Control (2017). Malaria strategic plan 2017–2022.

[7] Maina I, Wanjala P, Soti D, Kipruto H, Droti B, Boerma T (2017). Using health-facility data to assess subnational coverage of maternal and child health indicators. Kenya. Bull World Health Organ.

[8] WHO. Analysis and use of health facility data: guidance for malaria programme managers. Geneva, World Health Organization, 2018. https://www.who.int/healthinfo/tools_data_analysis_routine_facility/en/.

